# A Multiscale Point-Supervised Network for Counting Maize Tassels in the Wild

**DOI:** 10.34133/plantphenomics.0100

**Published:** 2023-10-02

**Authors:** Haoyu Zheng, Xijian Fan, Weihao Bo, Xubing Yang, Tardi Tjahjadi, Shichao Jin

**Affiliations:** ^1^ College of Information Science and Technology, Nanjing Forestry University, Nanjing, China.; ^2^ University of Warwick, Coventry, West Midland, UK.; ^3^ Nanjing Agriculture University, Nanjing, China.

## Abstract

Accurate counting of maize tassels is essential for monitoring crop growth and estimating crop yield. Recently, deep-learning-based object detection methods have been used for this purpose, where plant counts are estimated from the number of bounding boxes detected. However, these methods suffer from 2 issues: (a) The scales of maize tassels vary because of image capture from varying distances and crop growth stage; and (b) tassel areas tend to be affected by occlusions or complex backgrounds, making the detection inefficient. In this paper, we propose a multiscale lite attention enhancement network (MLAENet) that uses only point-level annotations (i.e., objects labeled with points) to count maize tassels in the wild. Specifically, the proposed method includes a new multicolumn lite feature extraction module that generates a scale-dependent density map by exploiting multiple dilated convolutions with different rates, capturing rich contextual information at different scales more effectively. In addition, a multifeature enhancement module that integrates an attention strategy is proposed to enable the model to distinguish between tassel areas and their complex backgrounds. Finally, a new up-sampling module, UP-Block, is designed to improve the quality of the estimated density map by automatically suppressing the gridding effect during the up-sampling process. Extensive experiments on 2 publicly available tassel-counting datasets, maize tassels counting and maize tassels counting from unmanned aerial vehicle, demonstrate that the proposed MLAENet achieves marked advantages in counting accuracy and inference speed compared to state-of-the-art methods. The model is publicly available at https://github.com/ShiratsuyuShigure/MLAENet-pytorch/tree/main.

## Graphical abstract

**Figure:**
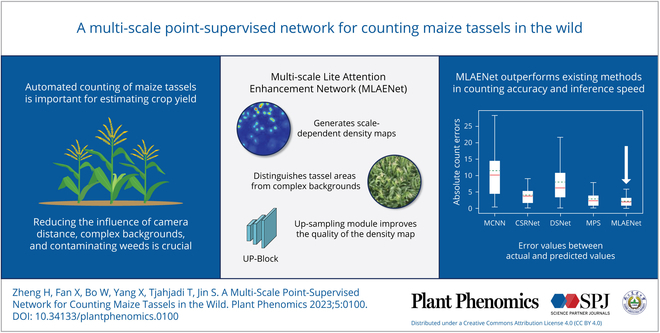

## Introduction

Maize is a widely cultivated cereal crop that serves as a staple food for a significant proportion of the global population. Accurate estimation of maize yield is important in enhancing crop production management, regulating the grain market, and ensuring food security [1]. Achieving this objective necessitates the precise counting of maize tassels, an indispensable step for farmers to estimate the potential yield of their crop. Therefore, developing an accurate and efficient maize tassel counting method is essential for improving the yield estimation and management of maize production.

Accurate counting of maize tassels also has broad implications. It plays a crucial role in research and development of new agricultural technologies [2], enabling researchers to evaluate different maize varieties and identify traits that contribute to higher yields. It helps monitor the impact of environmental factors on maize growth and development, aiding the development of strategies to mitigate effects of climate change and to optimize crop management practices. It also contributes to precision agriculture by allowing farmers to tailor inputs to the specific needs of the crop, reducing waste, and improving efficiency.

Manual counting of maize tassels used to be the only available method [3]. However, this method is time-consuming and labor-intensive, making it impractical when dealing with large fields or when repeated measurements are required. Furthermore, manual counting is subjective and prone to errors, as it heavily relies on the skill and experience of the person performing the counting.

A few methods based on traditional image processing [4,5] and machine learning [6–8] have been proposed for detecting and counting crops. Image-processing-based methods involve detecting and analyzing images of maize plants captured by cameras, enabling automated counting of the total number of plants and their approximate growth distribution based on the extracted features. However, such methods tend to be influenced by external environmental factors, e.g., interference due to weed, light reflection, and similar backgrounds, which present significant challenges in achieving accurate count. Machine-learning-based methods extract shallow features such as color, shape, and texture to detect and count crops [9].

With the rapid development of deep learning, deep convolutional neural networks (CNNs) have made unprecedented progress in computer vision algorithms for agriculture such as disease recognition and detection [10,11,45], crop land segmentation [12], and weed mapping [14]. CNNs have also been applied for counting tasks in crops and plants, which can be categorized into detect-and-count (DC), direct counting regression (DR), and density map estimation. DC methods start by segmenting the tassels from the input RGB (red–green–blue) images, followed by detecting each tassel based on the segmentation results and generating a bounding box around it [13,15–17]. However, DC methods are only suitable for images captured at close distances, resulting in small observation areas. In addition, labeling images with bounding boxes is a time-intensive task that becomes particularly challenging in densely distributed maize tassel fields due to significant occlusions. DR methods [18,19] predict a count map based on a smaller receptive field of the regression network containing redundant counts. This method avoids the annotation problem of DC because only point annotation of the image is required, thus significantly reducing the required workload. However, the DR methods fail to visualize the spatial distribution of the target objects. The density map estimation method [20] adopts density maps as the regression target, requires an RGB image as input, and generates a density map of the same size. The number of objects in the image is then obtained by integrating the density map. The generated density map provides a visual interpretation of the count results, and these methods can achieve excellent accuracy by generating high-quality density maps. Like DR, only point annotation is required. These advantages make generating density maps a promising approach to counting objects.

Currently, there are few studies that focus on counting maize tassels. Kumar [21] and Liu et al. [22] used a 2-stage network Fast R-CNN to count maize tassels with DC. Zan et al. [23] proposed integrating a random forest classifier to assist detection and classical CNN backbone VGG16 to deal with the complex background. Lu et al. [24] proposed TasselNet, which counts maize tassels by DR. Lu and Cao [25] proposed a contextual extension of TasselNet to achieve faster inference speed. However, all of the aforementioned methods use DC and DR to count maize tassels.

Because of its advantages, we are motivated to apply density map estimation-based method to the task of counting maize tassels. It only requires point labeling of dense maize tassels, which greatly reduces the manual labeling workload. The generated density map helps researchers to understand the spatial distribution of maize tassels. However, existing methods of generating density maps hardly show adequate performance in the task of maize tassel counting in the wild. Most density map estimation methods use Gaussian filtering to process the labeled map as ground truth. Multicolumn CNN (MCNN) [26] uses multiscale convolutional kernels to accommodate various object sizes, merging them to produce the density map. However, the shallow network structure might fail to learn a good representation of maize tassels of different shapes and thus limit the achievable accuracy. To achieve higher accuracy in object counting, Li et al. [27] proposed Dilated CNNs for Understanding the highly congested scenes (CSRNet), which uses a cascaded dilated convolutional structure. CSRNet achieves excellent performance in various object counting scenarios. However, its single-column structure is not well suited to address scale variation in maize tassel counting, and its back-end network with fixed dilated rate results in low inference speed. Stacked pooling (stack-pool) [28] addresses scale invariance via a stacked pool structure but lacks an efficient back-end network to aggregate and effectively utilize the extracted multiscale features. Dense scale network (DSNet) [29] uses cascading dilated convolutions with varying dilated rates to enhance feature fusion, but the single-column structure limits its accuracy in scenes with scale variations. Multitask point supervision (MPS) [30] uses 3 VGG16-based encoders to obtain 3 scales of feature maps, which are then aggregated in the dilated convolutional layer, improving the handling of scale variations. However, it performs poorly in fixed-scale counting scenarios, and its complex multicolumn structure results in low inference speed.

**Fig. 1.**
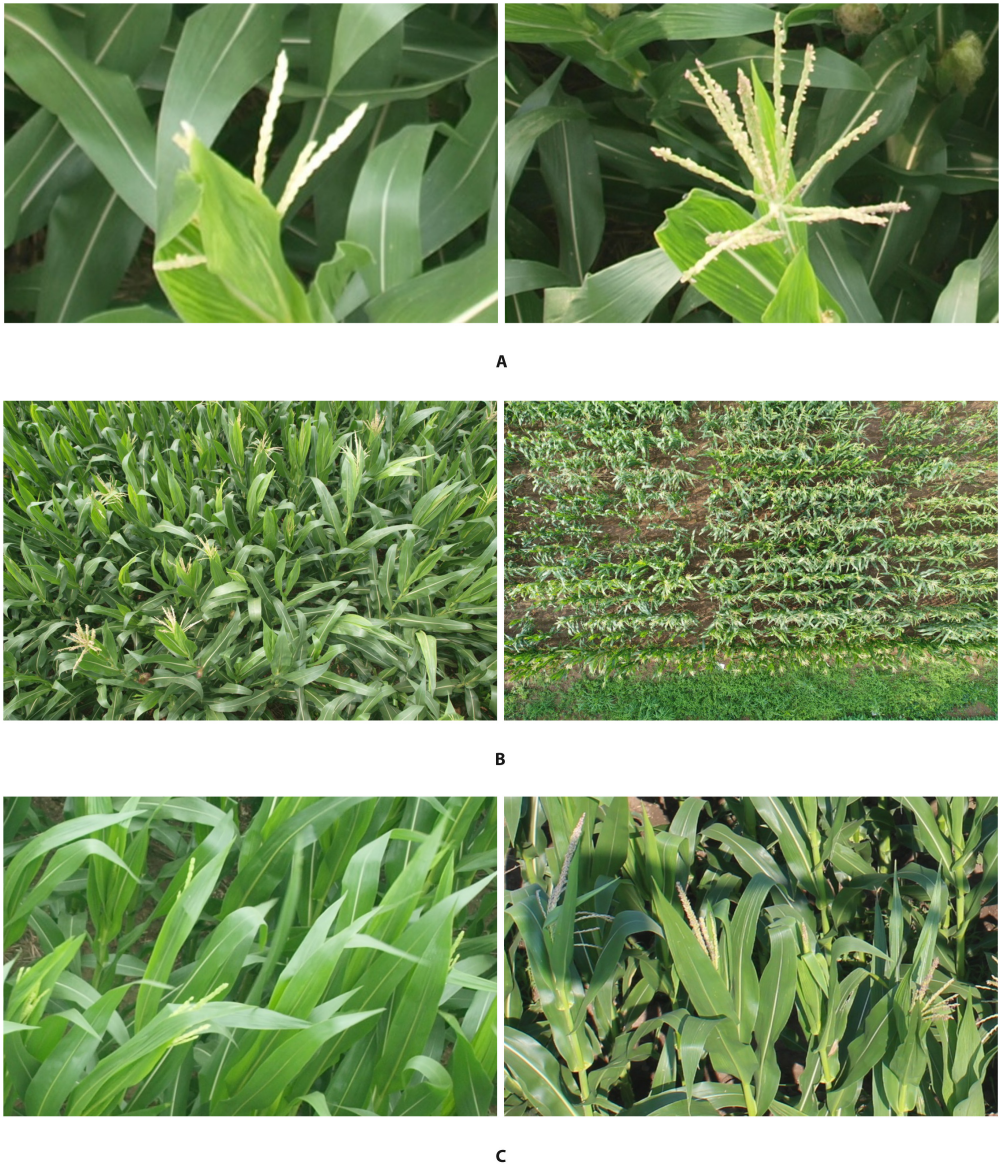
Appearances of maize tassels. (A) Diverse shapes and sizes at different stages of growth. (B) Different shapes and sizes when viewed at different distances from a fixed camera (left)/UAV (right). (C) Obscured by background disturbance in the field.

On the basis of the preceding analysis, direct application of density-map-based methods to the task of counting maize tassels may lose their validity due to 3 challenges that need to be addressed. First, maize tassels exhibit different sizes at various stages of growth as shown in Fig. 1A, and image capture, i.e., shooting, at different distances from the maize tassels leads to significant variations in the appearance of the tassels as shown in Fig. 1B. These cause the tassels to assume disparate shapes and sizes. Second, maize tassels are typically located in a field with complex background that results in numerous occlusions that interfere with counting, as shown in Fig. 1C. Third, to achieve rapid inference while ensuring accuracy in the counting proves exceedingly challenging for existing networks, which struggle to reconcile these 2 tasks.

To address the challenge of scale variation in images of maize tassels, we propose a ` feature extraction network that comprises a multicolumn cascaded dilated convolutional structure. This structure, which offers a different receptive field for every column while preserving the resolution of the feature map, facilitates the optimal utilisation of visual context information for object counting and the production of superior density maps. The quality of the density map is heavily influenced by its resolution. By incorporating multiple columns of varying dilated rates, the cascaded dilated convolution is capable of handling scale variations and outperforms multiple pooling methods while maintaining resolution. In addition, a novel up-sampling module, UP-Block, is devised to accomplish feature aggregation at different scales in the previous layer and up-sampling through a series of stacked convolutional layers and interpolation operations. It considerably ameliorates the quality of the density map, especially in scenarios where maize tassels are densely distributed, with only a marginal increase in the number of model parameters. Our network effectively handles diverse target scales, producing high-quality density maps to achieve accurate maize tassel counting.

To address the challenge of complex background interference, we propose a multifeature enhancement module (MFEM) by integrating a normalization-based attention mechanism. Since obscured features are suppressed by emphasizing salient features, a normalization-based attention mechanism is used to measure the significance of features and increase their corresponding weights. This allows MFEM to improve network performance without introducing additional model parameters, thus maintaining counting efficiency. Furthermore, the capability of MFEM to suppress background noise enables our network to generate more accurate density maps.

To balance efficiency and precision, we adopt an incremental dilated rate approach that increases the rate of dilation as the network width decreases. This strategy mitigates the slowdown caused by dilated convolution while maintaining a wide perceptual field and avoiding gridding effects. As a result, the proposed network achieves both high accuracy and speed. To reduce model complexity and facilitate its deployment, we design the lite feature extraction module (LFEM), to replace traditional convolution layers with lite convolution block (LCB). LCB leverages 1 × 1 convolution to downscale and stitch feature maps, thus significantly reducing the number of model parameters and computational effort. It also uses short connection to enhance information reuse. It ensures smooth information flow in the back-end network and prevents model degradation caused by increasing depth.

We succinctly summarize our major contributions as follows:

1. We propose an efficient multiscale lite attention enhancement network (MLAENet) for counting maize tassels based on input images. The network is capable of adapting to variations in maize tassel scales resulting from varying shooting distances and achieving excellent background suppression.
2. We design a multicolumn LFEM to adopt multiple dilated convolutions to capture wider contextual information at multiple scales in tassel images while reducing model complexity.
3. We design an MFEM to capture features at different scales. By integrating an attention mechanism, it focuses more on the target maize tassel features while mitigating the impact of complex background.
4. We propose a new up-sampling module, UP-Block, to complete the feature aggregation and up-sampling with small increase in the number of model parameters, which effectively improves the quality of the generated density maps.

## Materials and Methods

The images of the maize tassels in the field are obtained using a fixed camera or a camera mounted on UAV and simultaneously transferred to the MLAENet that generates density maps corresponding to the captured images. The camera (fixed or mounted on UAV) is switchable to continuously capture images of different areas, and the images are ceaselessly fed to MLAENet to obtain real-time density map predictions, which cannot be achieved by existing high-precision counting networks.

### Main framework of MLAENet

**Fig. 2.**
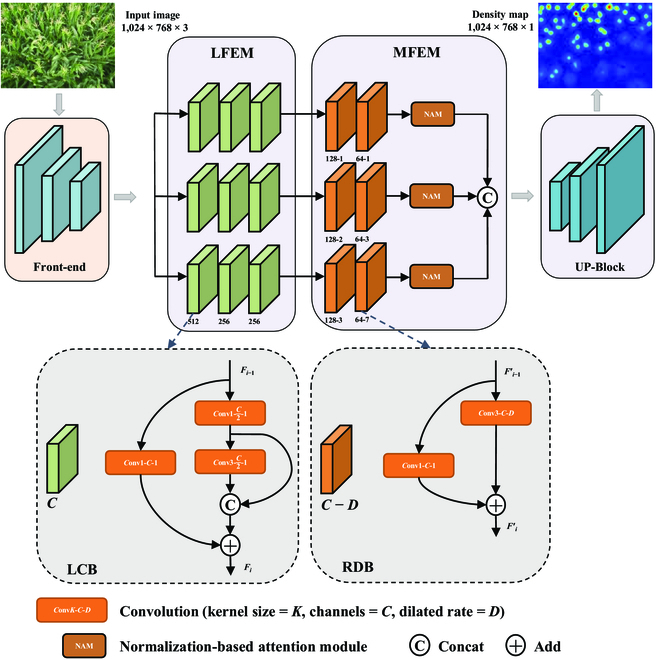
The detailed structure of MLAENet.

The basic structure of MLAENet is composed of 4 components: front-end, LFEM, MFEM, and UP-Block. Figure 2 shows the framework of the MLAENet for maize tassels counting (MTC).

We use the first 13 layers of VGG16 (fine-tuned) [31] as the front-end of our model. The model consists of many convolutional and pooling layers stacked on top of each other, forming a deeper network structure. VGG16 is a single-column structured front-end network that has strong generalization ability [27]. For the input image *In*, the front-end outputs its feature map *F*_0_ as given by

$$F_{0}=F_{\mathrm{vgg}}\left(\text{In}\right).$$ (1)

To facilitate the extraction of features at various scales and mitigate the potential negative impact of increasing model depth, it is necessary to obtain higher-level semantic information. To this end, we propose LFEM, a multicolumn structure that enables the extraction of more complex features. Each column of LFEM comprises 3 LCBs in cascade, producing 3 sets of feature maps ($F_{3}^{l}$) with deeper semantic information. To extract features at different scales from the generated feature maps, we introduce MFEM, which consists of residual dilated blocks (RDBs) with different dilation rates. Each column of MFEM contains 2 RDBs in cascade, generating feature maps with different receptive field sizes that contain various scales of contextual information. To further enhance salient features and mitigate the negative effects of complex background interference, we use normalization-based attention module (NAM) after the cascaded RDBs. The generated feature maps is concatenated along the channel dimension, and UP-Block is utilized for feature aggregation to generate the final density map.

### Lite feature extraction module

It is necessary to extract deeper semantic information for better extraction of features at different scales. A common strategy to do so is to increase the depth of the network by stacking convolutional layers to enhance the feature extraction capabilities of the model. However, blindly increasing the depth of the network poses a risk of model degradation and increases the model complexity. To mitigate the risk and increase in complexity, we introduce LCB to replace normal convolutional layers for feature extraction and compress the number of parameters in the model.

In LCB with width *C*, the channels of the input feature map are initially downscaled to $\frac{C}{2}$ using a 1 × 1 convolution kernel, followed by convolving the downscaled feature map using 3 × 3 convolution. This strategy results in a substantial reduction in computational effort compared to direct 3 × 3 convolution [32]. Nonetheless, it is imperative to retain the richness and redundancy of the features to ensure a comprehensive understanding of the input data. To preserve the expressiveness of the features, we combine the feature map after 1 × 1 convolution with the feature map after 3 × 3 convolution to leverage the redundant features as compensation for reducing the width of the 3 × 3 convolution kernel. Furthermore, we introduce the skip connection structure to further augment feature reuse and tackle the network degradation problem caused by increasing network depth. This structure effectively mitigates the gradient disappearance and network degradation problems by incorporating features from the previous convolutional layer. This structure helps facilitate the smooth flow of information throughout the back-end network. The computation of the *i*th LCB output *F_i_* for the input feature map *F*_*i*−1_ from the previous layer can be expressed by

$$F_{i}={\mathrm{Cov}}_{3,1}^{\frac{C}{2}}\left({\mathrm{Conv}}_{1,1}^{\frac{C}{2}}\left(F_{i-1}\right)\right)©{\text{Conv}}_{1,1}^{\frac{C}{2}}\left(F_{i-1}\right)⊕{\mathrm{Conv}}_{1,1}^{C}\left(F_{i-1}\right),$$ (2)

where ${\mathrm{Conv}}_{K,D}^{C}$ represents a convolutional layer with output channels *C*, kernel size *K*, and dilation rate *D*; *c* denotes channel-wise concatenation of feature maps; and ⊕ denotes element-wise addition of feature maps. Finally, the LFEM is composed of 3 columns of LCBs in cascade, and the output of LFEM *F_L_* is given by

$$F_{L}=\left(F_{3}^{1},F_{3}^{2},F_{3}^{3}\right),$$ (3)

where $F_{3}^{x}$ represents the output feature map of the third LCB in the *x*th column.

### Multifeature enhancement module

Using dilated convolution instead of pooling improves the preservation of spatial information and increases the receptive field. Thus, a cascaded dilated convolution maintains the resolution of the feature map while obtaining a larger receptive field. However, the normal single-column structure cannot cope well with the scale variation in maize tassel counting scenario. Therefore, we propose an MFEM to capture maize tassel features with different scales and highlight the target maize tassel features by integrating attention strategy. MFEM includes RDB and NAM. The former is designed to capture features with different scales, and the latter aims to cope with environmental interference by enhancing the weights of salient features.

#### Residual dilated block

The RDB processes the input feature map using dilated convolution and 1 × 1 convolution and adds them together. The use of 1 × 1 convolution aims to enhance the information reuse capability of the network. The computation of the *i*th RDB for the input feature map $F_{i-1}^{\prime }$ from the previous layer is given by

$$F_{i}^{\prime }={\mathrm{Conv}}_{3,D}^{C}\left(F_{i-1}^{\prime }\right)⊕{\mathrm{Conv}}_{1,1}^{C}\left(F_{i-1}^{\prime }\right).$$ (4)

It should be noted that the input feature map $F_{0}^{\prime }$ of the first RDB is equal to *F*_3_, which is the output feature map of the last LCB.

In counting the maize tassels, the decreasing size of the feature map causes some pixel-level information loss, which has a negative impact on the counting accuracy. Down-sampling through pooling causes a loss of pixel-level semantic information, as it reduces the resolution of the feature map. Using convolutional layers with larger kernel sizes may seem like a simple solution, but it significantly increases computational complexity and is not conducive to increasing model depth. Dilated residual networks [33] applies dilated convolution to perform semantic segmentation, and dilated convolution has a larger receptive field than normal convolution. The actual receptive field *R* of a dilated convolutional layer is given by

$$R=K+\left(K-1\right)*\left(D-1\right),$$ (5)

where *K* denotes the kernel size of the dilated convolutional layer.

The receptive field for cascaded dilated convolutions is given by

$$r_{n}=r_{n-1}+\left(R_{n}-1\right)\prod_{n=1}^{n-1}‍s_{i},$$ (6)

where *r_n_* is the perceptual field size of the layer, *R_n_* is the size of actual coverage of the layer (where the dilation rate needs to be considered for the dilated convolution), and *s_i_* is the step size of the *i*th layer. In our model, the stride of all convolutional layers is set to 1, and the kernel size of dilated convolution is set to 3. This gives the receptive field of the cascaded convolution in LFEM and MFEM relative to the input feature map *F*_0_ from the front-end as shown in Table 1.

**Table 1.** Receptive fields of the cascaded dilated convolutional layers.

	The first column					The second column					The third column				
Layer	1	2	3	4	5	1	2	3	4	5	1	2	3	4	5
Dilation rate	1					1	1	1	2	5	1	1	1	3	7
Receptive field (*n* × *n*)	3	5	7	9	11	3	5	7	11	21	3	5	7	13	27

**Fig. 3.**
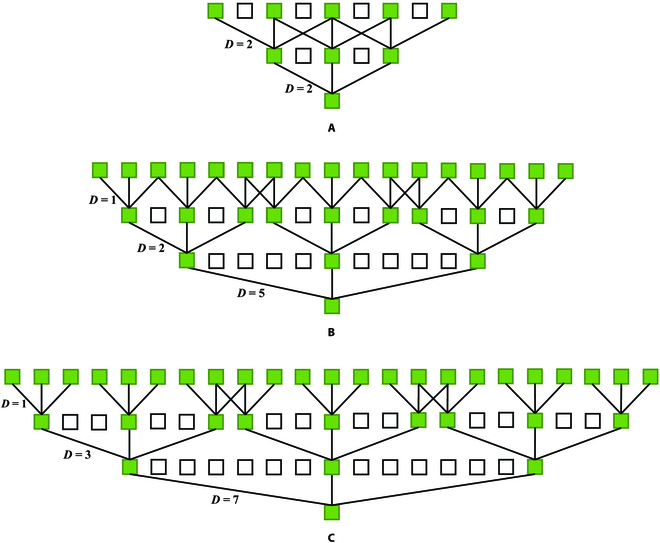
(A) A gridding effect occurs when the dilated rates are all set to 2, with half of the pixels not involved in the calculation. (B) At the dilated rate of [1,2,5], all pixels participate in the calculation, and no gridding effect occurs. (C) Dilated rate of [1,3,7] with no gridding effect.

Combining cascaded dilated convolution with different receptive fields improves the ability of the network to count maize tassels of different sizes. However, the fixed dilated rate design scheme results in slow inference speed and the gridding effect as shown in Fig. 3A. The gridding effect [34] refers to the phenomenon that occurs when dilated convolution is computed in a tessellation-like format, which leads to independent sets of convolution results from each layer, no interdependencies between these sets, and a loss of local information. This is problematic for pixel-level counting. Furthermore, although using cascaded dilated convolution does not increase the number of model parameters, it leads to cache-miss problems and slower inference because of discontinuous memory access and multiple data accesses during computation. To address these challenges and eliminate the gridding effect, we propose a revised dilated rate scheme. The scheme gradually increases the dilated rate as the number of channels decreases. By following this design principle, the negative impact of using dilated convolution on inference speed is minimized. The dilated rate of the first column is set to 1, while the remaining 2 columns have dilated rates of [1,2,5] as shown in Fig. 3B and [1,3,7] as shown in Fig. 3C. Because of adherence to the hybrid dilated convolution (HDC) [35], the structure effectively captures information from all pixels without causing the gridding effect. In contrast, the fixed dilated rate design scheme results in half of the pixels not being involved in the computation, as shown in Fig. 3A, which has a negative impact on counting accuracy. Simultaneously, lower dilated rates are used in the wider layers to mitigate the adverse effects of dilated convolution on the inference speed to a significant extent. With our proposed dilated rate design scheme, a desirable trade-off between speed and accuracy is achieved.

#### Normalization-based attention module

In neural network processing, the attention mechanism is used to enhance the efficiency of the network by selectively processing significant input data. There are 2 types of attention mechanisms used in neural networks: soft attention [36] and hard attention [37]. Soft attention involves calculating a weighted average of *N* inputs, while hard attention involves selecting only one input sequence at a specific position in the column, often based on the highest probability or randomly. Soft attention mechanisms are more widely used in CNNs [38,39] and can be further divided into the spatial, channel, and mixed domain attention mechanisms.

**Fig. 4.**
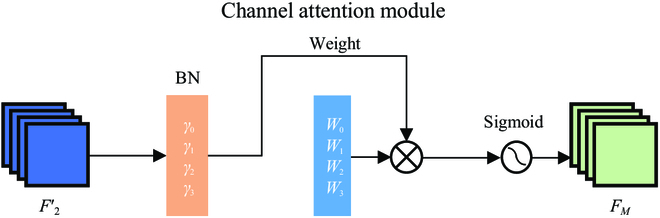
Structure of NAM.

Recent researches [40,41] have successfully incorporated attention mechanisms into counting tasks to accentuate significant features and minimize background interference. We implement the channel attention strategy of the NAM [42] due to its simplicity and effectiveness. NAM injects the attention map along the channels on an intermediate feature map in the CNN. The scaling factor of batch normalization (BN) is used to measure the variance of the channels and determine their significance, i.e.,

$$B_{\text{out}}=\mathrm{BN}\left(B_{\text{in}}\right)=\gamma \frac{B_{\text{in}}-\mu_{B}}{\sqrt{\sigma_{B}^{2}+\epsilon }}+\beta ,$$ (7)

where *μ_B_* and *σ_B_* respectively denote the mean and standard deviation of minibatch *B*. *γ* and *β* are trainable transformation parameters, respectively, for scaling and offset. The structure of NAM is shown in Fig. 4 and espressed as Eq. 8, where $F_{2}^{\prime }$ represents the output features from the last RDB, *F_M_* represents the output features of NAM, and *γ* is the scaling factor for each channel.

$$F_{M}=\text{sigmoid}\left(W_{\gamma }\left(\mathrm{BN}\left(F_{2}^{\prime }\right)\right)\right).$$ (8)

The weights are defined as

$$W_{\gamma }=\frac{\gamma_{i}}{\sum_{j=0}‍\gamma_{j}}.$$ (9)

Finally, RDBs and NAMs from multiple cascaded columns form the MFEM. The output feature map *F_O_* of MFEM is formed by concatenating the output feature maps *F_M_* of the NAMs in each column along the channel dimension, i.e.,

$$\mathrm{Fo}=F_{M}^{1}©F_{M}^{2}©F_{M}^{3}$$ (10)

### UP-Block

**Fig. 5.**
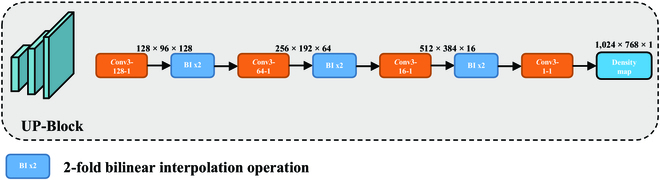
Structure of UP-Block.

To obtain the final density map at the same resolution as the input image, up-sampling and feature aggregation are required. The commonly used counting model uses a combination of 1 × 1 convolution for dimension reduction and high-rate bilinear interpolation for up-sampling. This method is fast, but the direct high-magnification interpolation negatively affects the quality of the counting and the generated density map. Transpose convolution [43] is regarded as an excellent alternative to bilinear interpolation due to its ability to learn. However, it is less efficient and may introduce the gridding effect in the generated images. Thus, to generate accurate density maps while ensuring efficiency, we design a lightweight up-sampling structure, UP-Block, as shown in Fig. 5. UP-Block consists of alternating 3 × 3 convolution and 2-fold bilinear interpolation layers. The purpose of using convolutional layers is to aggregate the features with different scales. The structure enables feature aggregation during the up-sampling process. The learning ability of the convolutional layer dramatically reduces the counting errors due to the up-sampling process, and the alternation of the convolutional layer and interpolation mitigates the gridding effect. The continuously decreasing number of channels limits the performance overhead of the convolutional layer to a low level. UP-Block achieves higher counting accuracy and generates higher-quality density maps with only a small increase in the number of model parameters.

### Loss function

We use Euclidean distance to measure the difference at the pixel level between the estimated density map and the ground truth, i.e.,

$$L\left(\Theta \right)=\frac{1}{2N}\sum_{i=1}^{N}‍{\left\|Z\left(X_{i};\Theta \right)-Z_{i}^{\mathrm{GT}}\right\|}_{2}^{2},$$ (11)

where *N* is the size of the training batch and *Z*(*X_i_*; Θ) is the output of MLAENet when the parameter is Θ. *X_i_* and $Z_{i}^{\mathrm{GT}}$ respectively denote the input image and its ground truth.

### Image acquisition

MTC [24] is a dataset for maize tassel counting. It contains 361 images of 6 maize varieties collected from 4 experimental fields in China. A charge-coupled device digital camera (E450 Olympus) was fixed at a position 4 or 5 m above the ground and acquired images at a tilted angle. The number of maize tassels in each camera image ranges from 0 to 100. The image resolutions are 3,456 × 2,304, 3,648 × 2,736, and 4,272 × 2,848. The resulting dataset is annotated with the position of each maize tassel in the form of point annotations.

**Fig. 6.**
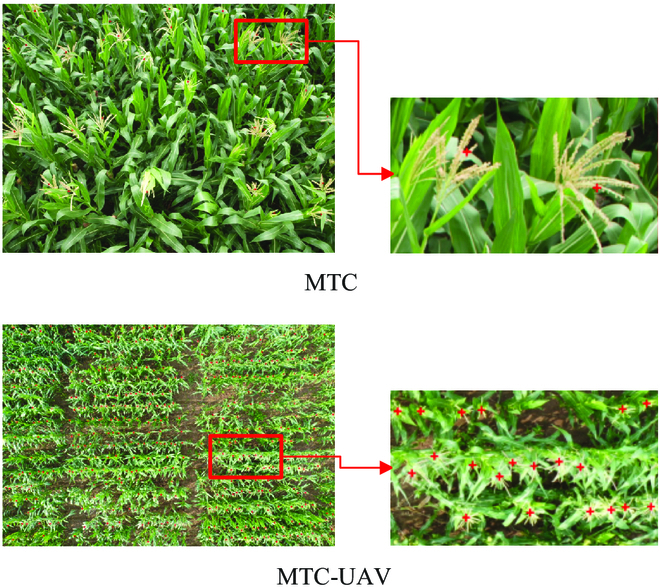
Sample annotated images from the MTC and MTC-UAV datasets.

MTC from unmanned aerial vehicle (MTC-UAV) dataset [44] is also used for maize tassel counting. The images were captured by a DJI Mavic 2 Pro UAV at a fixed altitude of 12.5 m above the ground of China Agricultural University. Over 400 maize varieties are included, with different types of maize randomly sown in plots of 5 m in length and 0.5 m in width. A total of 306 nonoverlapping images with a resolution of 5,472 × 3,648 are collected. The number of maize tassels in each image ranges from 36 to 550. The dataset is also annotated using point annotation. Sample annotations from the 2 datasets are shown in Fig. 6.

In this study, we resized the image resolution of MTC dataset to 1, 024 × 768 and MTC-UAV dataset to 1, 368 × 912 to optimize computational resources and expedite the training process. These resolutions are selected on the basis of the observation that no significant performance loss occurs when using them. Moreover, to improve network convergence speed and generalization and to reduce gradient disappearance, we normalized the input images. The datasets are then split into training, validation, and test sets with a ratio of 4:1:1. For the MTC dataset, there are 241 images for training, 60 for validation, and 60 for testing. For the MTC-UAV dataset, there are 204 images for training, 51 for validation, and 51 for testing. The training and validation sets are respectively used to train and evaluate the model, while the test set is used for the final model evaluation.

## Results

### Experimental design

All experiments were conducted on a single PC with Windows 11 as the operating system. The software environment for the experiment is PyTorch 1.11.0 with Cuda 11.6 and Python 3.8. The hardware is configured as Intel Core i7-13700KF @ 5.40 GHz, 32.0-GB memory, and NVIDIA GeForce RTX 3090Ti. We also used automatic mixed precision [45] in NVIDIA Apex to speed up our experiments. The opt level of automatic mixed precision is set to O1.

#### Training details

Gaussian filtering is used to generate density maps. This is achieved by treating maize tassels at pixel *x_i_* and using a function *δ*(*x* − *x_i_*), i.e., a function that is 1 while *x* = *x_i_* and 0 everywhere else. A graph with *N* maize tassels is

$$H\left(x\right)=\sum_{i=1}^{N}‍\delta \left(x-x_{i}\right).$$ (12)

To convert the graph to a continuous density function using a Gaussian kernel *G_σ_*, the density is

$$F\left(x\right)=H\left(x\right)*G_{\sigma }\left(x\right).$$ (13)

The propagation parameter *σ* needs to be determined according to the size of the maize tassels, which is related to the distance between the tassels at pixel *x_i_* and their neighboring maize tassels by observation. We adaptively determine the propagation parameters for each maize tassel based on the average distance between it and its neighbors. To prevent the propagation parameters from being too large in sparse scenarios and too small in dense scenarios, we impose restrictions on the minimum and maximum values of the propagation parameters. For each maize tassel *x_i_* in an image, we define the distance of that pixel point to its *k*th proximity domain as $\left\{d_{1}^{i},d_{2}^{i},\ldots ,d_{m}^{i}\right\}$. The average distance is

$$\bar{d^{i}}=\frac{1}{m}\sum_{j=1}^{m}d_{j}^{i}$$ (14)

The final density function is given by

$$\begin{array}{cc} F\left(x\right) & =\sum_{i=1}^{N}\delta \left(x-x_{i}\right)\;*\;G\sigma_{i}\left(x\right),\text{with}\\ \sigma_{i} & =\beta {\bar{d}}^{i},\sigma_{i}=\text{max}\left(\sigma_{i},\sigma_{\min}\right),\sigma_{i}=\text{min}\left(\sigma_{i},\sigma_{\max}\right) \end{array}$$ (15)

For MTC dataset, *β* = 0.06, *σ*_min_ = 15, and *σ*_max_ = 60. For MTC-UAV dataset, because of the fixed perspective, *σ* is set to 5.

To accurately evaluate the model performance, we adopt 5-fold cross-validation. The dataset is randomly split into 6 nonoverlapping subsets, with one subset used as the test set and the remaining 5 subsets used for model training and validation. The test set is fixed throughout the experiment, while the training and validation sets are split in a ratio of 4:1 from the 5 subsets. For each subset, we train multiple models on the training set and evaluate them on the validation set. We then select the best-performing model on the validation set and apply it to the corresponding test set, recording the performance metrics. We repeat this process until each subset is used as the validation set. Finally, we average the performance metrics of the 5 models on the 5 test sets to obtain the final model performance metrics.

In our experiments, all VGG16 are loaded with ImageNet [46] pretrained weights, and the other layers are initialized with Gaussian initialization with standard deviation of 0.1. We set the epoch number to 200 for MTC dataset and 300 for MTC-UAV dataset. The network is trained with a batch size of 4. Adaptive moment estimation (Adam) [47] is used as the learner because it demonstrates faster convergence than stochastic gradient descent with momentum. A fixed learning rate of 4 × 10^−6^ is used for training on MTC dataset, and 2 × 10^−6^ on MTC-UAV dataset. When calculating the inference speed of each model, we first perform a sufficient and equal-time warm-up of the graphics processing unit to ensure fairness of the results.

#### Evaluation metrics

Mean absolute error (MAE), root mean square error (RMSE), coefficients of determination (*R*^2^), and symmetric mean absolute percentage error (SMAPE) are used as evaluation metrics. MAE reflects the accuracy of the model, indicating the average deviation between the model predictions and the ground truth. RMSE reflects the robustness of the model, emphasizing larger errors and providing insights into the performance of the model in the presence of extreme values. SMAPE reflects the relative error between the model predictions and their ground truth, taking into account both overestimation and underestimation. SMAPE is scale-independent and can be used to compare different models or datasets. *R*^2^ reflects how well the model fits the ground truth, indicating the proportion of the variance in the dependent variable that can be predicted from the independent variable. The metrics are defined as follows:

$$\mathrm{MAE}=\frac{1}{N}\sum_{i-1}^{N}|C_{i}-C_{i}^{\mathrm{GT}}|,$$ (16)

$$\text{RMSE}=\sqrt{\frac{1}{N}\sum_{i-1}^{N}{\left|C_{i}-C_{i}^{\mathrm{GT}}\right|}^{2}}$$ (17)

$$\text{SMAPE}=\frac{100\%}{N}\sum_{i=1}^{N}\frac{{\left|C_{i}-C_{i}^{\mathrm{GT}}\right|}^{2}}{\frac{|C_{i}|+|C_{i}^{\mathrm{GT}}|}{2}},$$ (18)

$$R^{2}=1-\frac{\Sigma_{i=1}^{N}{\left|C_{i}-C_{i}^{\mathrm{GT}}\right|}^{2}}{\Sigma_{i=1}^{N}{\left|C_{i}-{\bar{C}}_{i}^{\mathrm{GT}}\right|}^{2}},$$ (19)

where *N* is the number of images in the test set, $C_{i}^{\mathrm{GT}}$ is the ground truth of counting, and ${\bar{C}}_{i}^{\mathrm{GT}}$ represents the mean value of $C_{i}^{\mathrm{GT}}$ in the test set. *C_i_* represents the count of the output density map, and its value is the sum of the values on each pixel of the output density map.

### Comparison with other methods

To ascertain the effectiveness of our proposed method, we conducted a comparative analysis with several state-of-the-art techniques, including MCNN, CSRNet, stack-pool, DSNet, and MPS on MTC and MTC-UAV datasets. All methods were trained under identical conditions, utilising the same data augmentation methods.

#### Advantages in counting accuracy

**Fig. 7.**
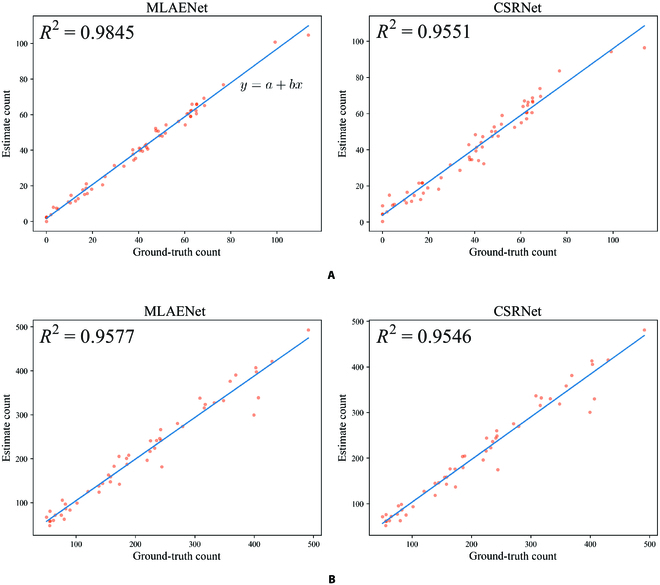
Coefficients of determination of the MLAENet and CSRNet on (A) MTC and (B) MTC-UAV datasets.

Achieving high counting accuracy in both scale-varying and scale-fixed scenarios is a formidable challenge. Nevertheless, our proposed method exhibits high accuracy in both scenarios. Table 2 shows that MLAENet model achieves outstanding performance. Its success in achieving high counting accuracy in diverse scenarios is attributed to its multicolumn structure, efficient feature enhancement, and aggregation techniques. The model outperforms other counting models on both datasets, with the lowest MAE, RMSE, and SMAPE scores. The MTC dataset, which exhibits scale-varying properties, is particularly challenging, and only MLAENet and MPS models achieve *R*^2^ > 0.97. This result emphasizes the importance of the multicolumn structure in addressing scale-varying scenarios. However, the MPS model performs worse on the scale-fixed MTC-UAV dataset compared to its performance on the scale-varying MTC dataset. On the other hand, the single-column structured CSRNet model does not exhibit any advantage over multicolumn structures on MTC dataset. This highlights the difficulty in achieving high counting accuracy under different image acquisition conditions. Despite MCNN also using a multicolumn structure, its shallow network design limits the effectiveness of its feature extraction. Similarly, the lack of an effective feature extraction method in the stack-pool model is also inadequate for accurate counting. This suggests that enhancing feature extraction and aggregating useful features at different scales are crucial for achieving high counting accuracy. The dense connection structure of DSNet model is not well suited for handling scale changes in maize tassel counting. Since these scale changes are discontinuous, the retention of a large number of features at different levels without relevant feature aggregation results in poorer performance than CSRNet on both datasets. This demonstrates the inadequacy of focusing solely on enhancing feature extraction and highlights the importance of enhancing useful features and aggregating them at different scales. The plots of coefficients of determination shown in Fig. 7 demonstrate that the overestimations and underestimations of MLAENet are stable compared to CSRNet, especially on MTC dataset. The calculation of the straight-line expression in Fig. 7 is as follows:

$$b=\frac{\sum_{i=1}^{N}‍\left(C_{i}^{\mathrm{GT}}-{\bar{C}}_{i}^{\mathrm{GT}}\right)\left(C_{i}-{\bar{C}}_{i}\right)}{\sum_{i=1}^{N}‍{\left(C_{i}^{\mathrm{GT}}-{\bar{C}}_{i}^{\mathrm{GT}}\right)}^{2}}$$ (20)

$$a={\bar{C}}_{i}-b{\bar{C}}_{i}^{GT}.$$ (21)

**Table 2.** Comparison of different networks on MTC and MTC-UAV datasets.

		MTC				MTC-UAV			
Method	Year, venue	MAE	RMSE	SMAPE	*R* ^2^	MAE	RMSE	SMAPE	*R* ^2^
MCNN	2016 CVPR	11.6	15.4	46.7%	0.2949	53.1	71.5	27.4%	0.4298
CSRNet	2018 CVPR	4.0	5.1	28.4%	0.9551	15.1	24.1	9.0%	0.9546
Stack-pool	2020 ICASSP	56.5	73.4	135.9%	0.0090	120.6	156.5	33.4%	0.0800
DSNet	2021 ICMR	8.0	10.3	37.7%	0.7586	43.4	50.7	27.0%	0.7008
MPS	2022 ICASSP	2.9	3.9	23.8%	0.9720	17.9	28.5	10.4%	0.9385
MLAENet	This paper	**2.3**	**3.1**	**23.3%**	**0.9845**	**14.8**	**23.5**	**8.7%**	**0.9577**

**Fig. 8.**
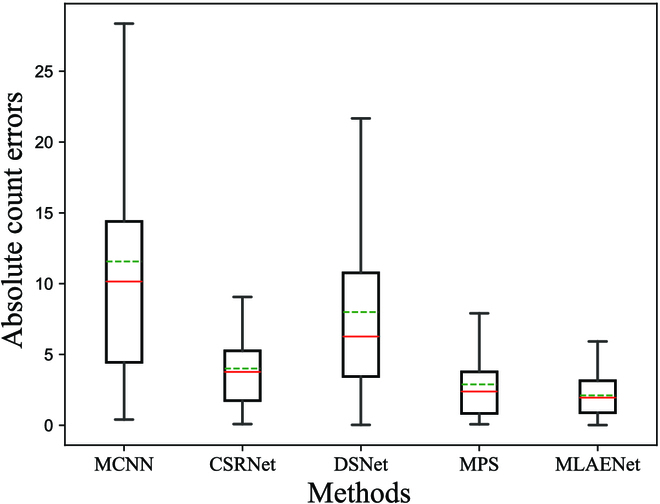
Box plots of error values between ground-truth and predicted values for different methods in dense scenarios with scale variation.

To offer a more intuitive representation of the counting performance of MLAENet in densely populated scenes with scale variation, we created boxplots illustrating the error values between the predicted and ground-truth counts for each sample in the MTC dataset. We compared various methods, including MCNN, CSRNet, DSNet, and MPS. The counting performance of stack-pool is excluded owing to its inferior results. As depicted in Fig. 8, the box plots convey the minimum and maximum errors through 2 short horizontal lines outside the box. The median and mean values are respectively denoted by the red and green lines, while the quartiles are illustrated by the 2 vertical black lines. At present, the findings demonstrate that MLAENet has the shortest box height, suggesting the most consistent data fluctuation. Moreover, MLAENet displays the smallest upper limit, indicating the lowest counting error. Consequently, MLAENet surpasses other methods in terms of efficiency and stability in densely populated scenes with scale variation.

#### Advantages in density map estimation

**Fig. 9.**
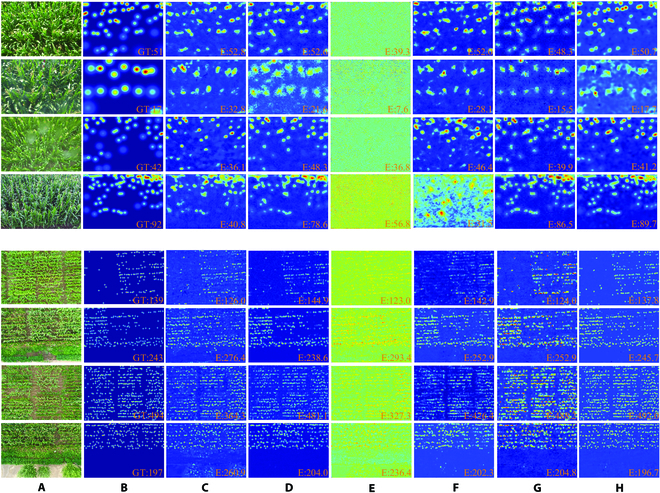
Inference density map on MTC (rows 1 to 4) and MTC-UAV (rows 5 to 8) dataset. The columns are (A) original image (annotated); (B) ground truth; and results using (C) MCNN, (D) CSRNet, (E) stack-pool, (F) DSNet, (G) MPS, and (H) MLAENet. GT, ground truth of count; E, estimated count.

Figure 9 shows the density map generation and counting results obtained by different networks on MTC and MTC-UAV datasets, respectively. Our proposed MLAENet accurately locates maize tassels while effectively distinguishing them from other plants. For example, in the third image of the MTC-UAV dataset (row 7 of Fig. 9), because of the large camera shooting distance and the concentrated distribution of maize tassels, many targets are incorrectly identified as other plants resulting in missed detections for other methods. In contrast, our model distinguishes between maize tassels and other plants, leading to more accurate counting results. This demonstrates the effectiveness of MFEM. In the fourth image of the MTC dataset (row 4 of Fig. 9), because of the shooting perspective, the maize tassels are concentrated in the top part of the image, resulting in severe occlusion. Our network accurately recognizes the features of the maize tassels and leverages the large receptive field to fully utilize contextual information, leading to more accurate counting results than other networks. In addition, our density map generated by MLAENet is significantly more accurate and smoother compared to those generated by other networks, as our network uses UP-Block for up-sampling instead of just using 1 × 1 convolution and direct interpolation.

**Fig. 10.**
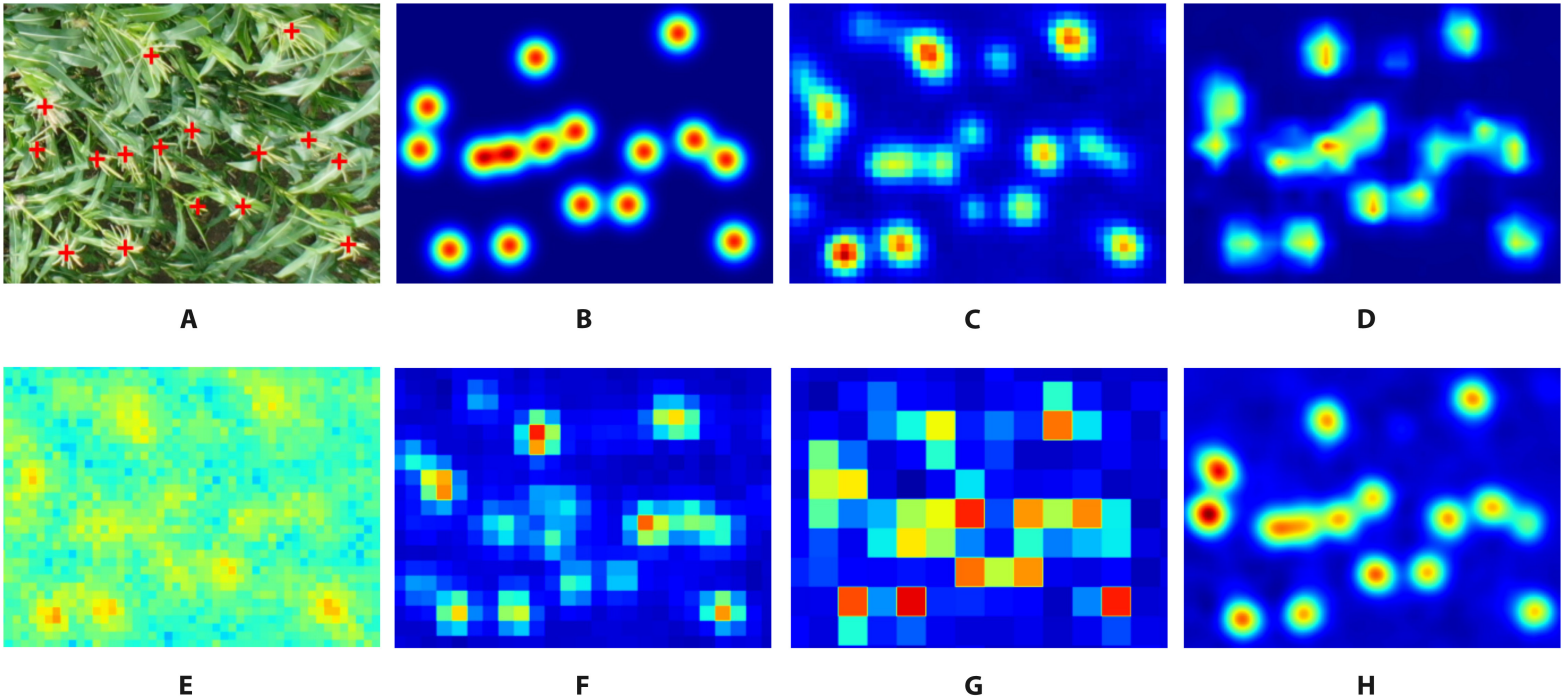
Inference density map when the maize tassels are densely distributed. (A) Original image (annotated); (B) ground truth; and results using (C) MCNN, (D) CSRNet, (E) stack-pool, (F) DSNet, (G) MPS, and (H) MLAENet.

The image in row 4 also illustrates the challenge of counting maize tassels in dense canopies, where the tassels are closely compacted and partially hidden by leaves and stems. All existing models have shown a larger counting error compared to the open-canopy scenario. However, our network shows some advantages in these dense canopies, but there is still room for further improvement. Because of the severe occlusion problem, dense canopies have high requirements for feature extraction and utilization capabilities of the model. Thus, we plan in the future to use more effective feature extraction methods to replace VGG16 and use infrared images to assist in counting to achieve higher accuracy in dense canopies. It is important to note that the smooth density map not only helps in locating maize tassels more accurately but also represents the density of maize tassels more precisely. As shown in Fig. 10, our network generates smooth density maps that reflect the spatial distribution of the tassels more precisely than other methods.

#### Advantages in inference speed and the parameters

To assess the inference speed of each network, we conducted tests using input images of resolution of 1, 024 × 768. To make a more robust comparison of the models, we evaluated their inference speed under the standard of qualified accuracy, i.e., when the network achieves *R*^2^ ≥ 0.9 on both MTC and MTC-UAV datasets. The results are presented in Table 3. While existing high-speed inference networks, such as MCNN, stack-pool, and DSNet, offer high inference speed, they are not optimized for the task of counting maize tassels in various environments, leading to low counting accuracy and inaccurate density maps. Achieving high counting performance with simple network structures poses a significant challenge. Some models, such as dilated convolution with an unreasonable dilated rate design scheme and multicolumn structure, often significantly reduce inference speed. The fixed dilation rate design scheme results in a slower inference speed of 10.14 frames/s (FPS), which is only one-third of the performance achieved by MLAENet.

**Table 3.** Comparing the parameters of different networks and their inference speed when inputting images with 1, 024 × 768 resolution. The networks with qualified accuracy are compared individually and ranked in ascending order of FPS.

Method	Venue, year	Parameter	FPS	Qualified
DSNet	2021 ICMR	20.69M	48.76	No
MCNN	2016 CVPR	0.13M	85.30	No
Stack-pool	2020 ICASSP	0.20M	54.45	No
MPS	2022 ICASSP	21.94M	5.11	Yes
CSRNet	2018 CVPR	16.26M	10.14	Yes
MLAENet	This paper	14.00M	32.90	Yes

Although MLAENet has only a slight advantage over MPS in accuracy, there is a significant disparity in inference speed between MLAENet and MPS. The multicolumn structure of MPS, which is based on different pooling levels, is a widely adopted approach for addressing scale variations in object counting and detection. This strategy enhances the capacity of the network to handle multiscale-scale features. However, it also introduces a higher computational cost to the backend network, as it requires the processing of feature maps with various pooling levels from the backbone. Many multiscale networks use this concept, but the backend network considerably slows down the inference speed when dealing with feature maps at smaller pooling levels (e.g., 2× and 4×). In the experiments, our multiscale feature extraction method, based on LCB and RDB, not only enhances the capacity of the network to handle multiscale features but also ensures efficient model inference speed, striking a favorable balance between accuracy and speed. In comparison to MPS, MLAENet achieves an impressive inference speed of 32.90 FPS, which is over 6 times faster than MPS. Thus, MLAENet is capable of real-time processing tasks, whereas MPS is not.

By incorporating LCB, a reduced number of model parameters is achieved (even fewer than CSRNet with a single-column structure) while maintaining high counting accuracy. Reducing the number of model parameters significantly decreases the storage demand of the model, making it simpler to deploy in resource-constrained environments such as mobile devices or embedded systems. Our model leverages an efficiently designed back-end network to strike a balance between speed and accuracy. In conclusion, our model achieves superior accuracy and inference speed performance, making it an effective solution for counting maize tassels in different scenarios.

### Ablation study

We demonstrate the effectiveness of several key parts of our designed network structure through their corresponding ablation experiment. The specific structure of the model after removing a certain module is as follows: When the LCB is replaced, a normal convolutional layer is used instead. When the RDB is replaced, a dilated convolutional layer is used instead. When the UP-Block is replaced, upsampling via a 1 × 1 convolutional layer and 8-fold bilinear interpolation layers are used instead.

#### The impact of different dilated rate design schemes

To investigate the impact of dilated rate design on the performance of multicolumn cascaded dilated convolution, we compared 3 design schemes while keeping the network structure unchanged. The first scheme utilizes normal convolutions instead of dilated ones, yielding the fastest inference speed. The second scheme uses a fixed dilated rate of 2 for all convolution layers, as in CSRNet. The third design scheme in our network features a carefully designed dilated rate for each convolution layer. We evaluated the accuracy and inference speed of these schemes on MTC and MTC-UAV datasets, with frames per second as the metric at a resolution of 1, 024 × 768. As shown in Table 4, replacing all dilated convolutions with normal convolutions led to the fastest inference speed. However, this is achieved at the expense of significant accuracy degradation. Conversely, using a fixed dilated rate of 2 results in a much slower inference speed. Our proposed dilated rate design scheme achieves a desirable trade-off between speed and accuracy. It outperforms the scheme used in CSRNet in terms of accuracy while achieving a significant speedup in inference. Although the inference speed achieved with our dilated rate design scheme is not as fast as that with normal convolutions, the considerable improvement in counting accuracy justifies the compromise in speed. Overall, our scheme strikes a good balance between accuracy and inference speed.

**Table 4.** Accuracy and speed performance of 3 different dilated rate design schemes on MTC dataset.

	MTC				MTC-UAV				
Design	MAE	RMSE	SMAPE	*R* ^2^	MAE	RMSE	SMAPE	*R* ^2^	FPS
All set 1	3.2	4.1	25.4%	0.9711	16.3	28.3	9.4%	0.9372	50.18
All set 2	2.6	3.4	23.5%	0.9802	15.5	27.6	8.9%	0.9401	12.64
Ours	2.3	3.1	23.3%	0.9845	14.8	23.5	8.7%	0.9577	32.90

#### Benefits from NAM and UP-Block

**Fig. 11.**
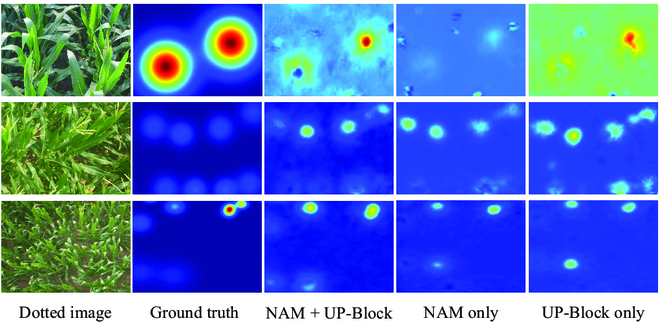
Example of ablation experiment results. Target loss is observed after the removal of UP-Block, and significant background interference occurs after the elimination of NAM.

We conducted experiments to evaluate the impact of removing NAM and UP-Block modules on the performance of our network on MTC and MTC-UAV datasets. The results are presented in Table 5. Our analysis indicates that NAM is a critical component of MFEM as it highlights important features while suppressing less significant ones. This leads to improved accuracy in counting maize tassels, which is challenging because of complex and varying background disturbances. Removing NAM reduces the weight of useful features, resulting in a weakened ability to handle environmental disturbances and decreased counting accuracy. In addition, NAM based on the channel attention mechanism steadily improves the accuracy of the model without increasing its complexity, making it an efficient approach for feature selection. Besides, the UP-Block module plays a crucial role in feature aggregation and up-sampling, significantly improving the performance of the model. Removing the UP-Block module causes a noticeable decrease in accuracy on MTC dataset, as direct interpolation fails to aggregate features. By using convolutional layers in the up-sampling module, our model achieves better feature aggregation and, consequently, better performance. Furthermore, removing the UP-Block module adversely affects the quality of the density maps, causing occasional misdetection of count targets. The experimental results in Fig. 11 show that the best performance is achieved when both NAM and UP-Block module are used.

**Table 5.** Ablation study experiment on MTC and MTC-UAV datasets.

					MTC				MTC-UAV			
NAM	UP-Block	LCB	RDB	Parameter	MAE	RMSE	SMAPE	*R* ^2^	MAE	RMSE	SMAPE	*R* ^2^
	√	√	√	14.00M7	2.8	3.6	25.5%	0.9767	15.2%	25.7	8.7%	0.9493
√		√	√	13.78M	2.9	3.7	25.0%	0.9778	16.7	27.9	9.6%	0.9386
√	√		√	21.44M	5.0	6.2	34.6%	0.9363	18.3	30.4	12.9%	0.9237
√	√	√		13.88M	2.6	3.3	24.7%	0.9807	15.5	26.8	9.1%	0.9426
√	√	√	√	14.00M	2.3	3.1	23.3%	0.9845	14.8	23.5	8.7%	0.9577

#### Benefits from LCB

The results presented in Table 5 demonstrate the crucial roles of LCB in the model. The use of LCB reduces the number of model parameters by 34.7%, while simultaneously improving its counting accuracy. Furthermore, the use of 1 × 1 convolution effectively reduces the number of model parameters used in the layers with larger widths. Moreover, the introduction of skip connections not only helps alleviate the problem of vanishing gradients but also enables the utilization of front-layer feature maps. This facilitates information flow in the network and leads to an improvement in the performance of the model. By replacing conventional convolutional layers with LCB, we ensure that the network is more deployable while maintaining high counting accuracy. This significantly enhances the practicality of our network.

## Conclusion

This paper introduces MLAENet, a novel model for counting maize tassels and generating high-quality density maps. It tackles scale-varying scenarios with 2 multicolumn modules: LFEM and MFEM. LFEM utilizes LCB to reduce model complexity, while MFEM uses RDB and NAM to enhance accuracy. UP-Block aggregates features to generate precise density maps.

MLAENet has been tested on 2 datasets: the MTC dataset with fixed camera and varying scales and the MTC-UAV dataset with UAV and fixed scales. It demonstrates robust performance in different scenes, achieving an *R*^2^ greater than 0.98 on the MTC dataset. It also exhibits a fast inference speed of 32.90 FPS on a single RGB image of 1, 024 × 768 resolution, outperforming other models. MLAENet surpasses state-of-the-art models, particularly in scale-varying scenarios, with high accuracy and fast inference speed. However, it still uses the older VGG16 as front-end. Future work will explore more advanced and efficient feature extraction tools to further enhance the network performance.

## Data Availability

The data that support the findings of this study are available from http://doi.org/10.1186/s13007-017-0224-0 (MTC dataset) and https://doi.org/10.1109/TGRS.2021.3058962 (MTC-UAV dataset).
